# *PsPRE1* is a basic helix-loop-helix transcription factor that confers enhanced root growth and tolerance to salt stress in poplar

**DOI:** 10.48130/FR-2023-0016

**Published:** 2023-06-29

**Authors:** Jiujun Du, Xiaolan Ge, Hantian Wei, Min Zhang, Yongxia Bai, Lei Zhang, Jianjun Hu

**Affiliations:** 1 State Key Laboratory of Tree Genetics and Breeding, Key Laboratory of Tree Breeding and Cultivation of the National Forestry and Grassland Administration, Research Institute of Forestry, Chinese Academy of Forestry, Beijing 100091, China; 2 Collaborative Innovation Center of Sustainable Forestry in Southern China, Nanjing Forestry University, Nanjing 210037, China

**Keywords:** Poplar, *PsPRE1*, Adventitious root, Quantitative trait locus, Salt stress, Hormone

## Abstract

The basic helix-loop-helix (bHLH) family of transcription factors is one of the largest and oldest transcription factor families in plants. Members of the bHLH family regulate various growth and metabolic processes in plants. We used quantitative trait locus (QTL) mapping and transcriptome sequencing (RNA-seq) to identify *PRE1* as a candidate bHLH transcription factor associated with root dry weight (RDW) in poplar. *PRE1* was highly expressed in the roots and xylem, and was responsive to gibberellin, salicylic acid, drought, and salt stress. We cloned the *PRE1* homolog from *Populus simonii* 'Tongliao1', referred to as *PsPRE1*, and transformed it into 84K poplar (*Populus* *alba* × *Populus* *glandulosa*). The overexpression of *PsPRE1* in 84K poplar increased adventitious root development, fresh weight, total root number, and maximum root length. Poplar lines overexpressing *PsPRE1* also exhibited enhanced salt tolerance while retaining a normal growth phenotype in the presence of salt stress. Catalase (CAT) activity in the *PsPRE1* overexpression lines was higher than that of the wild-type, which may play a role in detoxifying stress-induced hydrogen peroxide production. An RNA-seq analysis of the *PsPRE1* overexpression line revealed several differentially expressed genes (DEGs) involved in or related to auxin-, gibberellin-, and salicylic acid pathways, which indicates that the regulation of root development in poplar by *PsPRE1* may involve multiple hormones.

## Introduction

Roots are essential plant organs that serve critical functions such as nutrient and water absorption, hormone synthesis, storage, and mechanical support^[1−4]^. Their close association with the soil enables roots to quickly sense the onset of environmental stresses such as drought and salinity^[2, 3]^. Early environmental stress detection is crucial for activating appropriate physiological responses that enable the plant to tolerate and potentially adapt to these stresses.

Studies have shown that basic helix-loop-helix (bHLH) transcriptional regulators play a key role in plant root development. The MYB-WD40-bHLH (MWB) complex regulates the expression of *GLABRA2* (*GL2*) and *CAPRICE* (*CPC*) and thereby directs the development of plant root hairs^[3]^. GL2 binds directly to the upstream promoter region of *ROOT HAIR DEFECTIVE6* (*RHD6*), *ROOT HAIR DEFECTIVE 6-LIKE1* (*RSL1*), *RSL2*, Lj*-RHL-LIKE1* (*LRL1*), and *LRL2* to regulate plant root hair development^[5−8]^. Myelocytomatosis (MYC) is a bHLH family member that promotes lateral root growth and inhibits adventitious root and primary root development during normal development and in response to stress^[9]^. *Populus simonii × P. nigra* *PxbHLH02* is another bHLH gene and *Populus simonii × P. nigra* overexpressing *PxbHLH02* promotes root elongation under stress and promotes greater resilience to stress^[10]^. Gibberellin is another plant hormone that also regulates root development^[11−14]^. A bHLH protein CESTA (CES) induces gibberellin 2-oxidase *GA2ox7* to inhibit root elongation^[15]^. Furthermore, recent research has indicated that bHLH transcription factors are also involved in the regulation of salt stress responses. Overexpression of *AtbHLH122* and *Oryza rufipogon* *OrbHLH001* confers salt tolerance in plants, which is achieved by increasing proline levels and improving ROS scavenging capacity^[16−18]^.

The bHLH superfamily of transcriptional regulators is an ancient and conserved protein family characterized by two conserved domains: the basic region at the N-terminus (10−20 amino acids in length) and the helix-loop-helix (HLH) domain at the C-terminus (20−40 amino acids in length)^[19−21]^. The HLH domain is the structural basis for the formation of dimers and regulatory functions of the proteins in this family^[22]^. The bHLH superfamily can be divided into different types based on the amino acid residues present in the basic region. Glu-13 and Arg/Lys-16 are essential for binding to the E-box cis element (5'-CANNTG-3') found in the promoters^[19, 23]^ of stress-related genes such as *AtbHLH112*, *AtbHLH122*, and *NtbHLH123*^[16, 18, 24]^. G-box (5'-CACGTG-3) is a sub-type of E-box cis element. His/Lys-9, Glu-13, and Arg-17 in the basic region of bHLH proteins promotes binding to the G-box cis element^[19, 23, 25]^. E-box binding type bHLH can bind all E-box elements except G-box, while G-box binding sub-type can bind all E-box elements.

*PACLOBUTRAZOL RESISTANCE1* (*PRE1*) is a bHLH family gene that regulates several aspects of gibberellin-dependent responses, such as hypocotyl and petiole elongation in *Arabidopsis thaliana*^[26]^. Plant cell size is thought to be determined by a triagonistic balance between the bHLH proteins ACTIVATOR OF CELL ELONGATION (ACE) or CRYPTOCHROME INTERACTING BASIC HELIX-LOOP-HELIX5 (CIB5), ARABIDOPSIS ILI1 BINDING BHLH1 (IBH1), and PRE1. ACE and CIB5 are positive regulators of cell elongation that are inhibited by IBH1. AtPRE1 promotes cell elongation by binding to and inhibiting IBH1, which allows ACE or CIB5 to function and promote cell elongation^[27, 28]^. Similarly, HLH4 is involved in this process and functions similarly to IBH1 by inhibiting the expression of *EXPANSINS8* (*EXP8*), *EXP11*, *indole-3-acetic acid 7* (*IAA7*), and *IAA11* by binding to CIB5. PRE1 activates expression of these genes by binding to and inhibiting the function of HLH4^[28]^. Much is known about the role of *PRE1* genes in cell elongation, but studies on the involvement of *PRE1* genes in plant responses to stress are lacking.

Deep roots and strong leaves complement each other, and well-developed roots are beneficial to the growth of the plant. Poplar is a woody model plant and an important energy tree species. It is a fast-growing tree with a large biomass accumulation in terrestrial ecosystems, extensively used in the pulp and paper industry, reforestation of land and bioenergy feedstocks^[29]^. This study reveals the positive effect of *PRE1* on adventitious root development and salt stress tolerance in poplar and provides a theoretical basis for the research and propagation of woody plants. Developing drought- and salt-tolerant poplar varieties will lead to increased growth and biomass accumulation in areas where these stresses are often encountered. The development of stress-tolerant poplar cultivars requires a better understanding of the molecular mechanisms behind drought and salt stress responses. By expanding the growing range of poplar into dryer and more saline environments, more poplar can be cultivated as an alternative energy source to help reduce the use of fossil fuels and protect the global environment.

## Materials and methods

### Plant materials and growth environment

*Populus deltoides* 'Danhong' ('Danhong'), *Populus simonii* 'Tongliao1' ('Tongliao1'), and two hybrid offspring (*Populus deltoides* 'Danhong' × *Populus simonii* 'Tongliao1') with opposite rooting types (F64 – good rooting (GR) offspring, F75 – bad rooting (BR) offspring)^[30]^ were selected and preserved at the Chinese Academy of Forestry. All plant material was cut into 15 cm cuttings with at least two buds and cultivated in the greenhouse at the Chinese Academy of Forestry and raised under natural light and temperature (20−25 °C).

84K poplar (*Populus* *alba* × *Populus* *glandulosa*) was used for genetic transformation and hormone treatments. Tissue culture seedlings were cultivated in the tissue culture room at the Chinese Academy of Forestry at 25 °C with a 16 h photoperiod at an intensity of 2,500 lux.

### Sequence alignment of the PsPRE1 protein with its homologs and construction of the phylogenetic tree

PsPRE1 homologs in diverse plant species (*Arabidopsis thaliana*, *Populus trichocarpa*, *Zea mays*, and *Oryza sativa*) were identified using the PsPRE1 full protein sequence with the E-value cutoff set to e-29 at the online site Phytozome (https://phytozome-next.jgi.doe.gov/). A phylogenetic tree was constructed using MEGA 11 (https://www.megasoftware.net/) with the neighbor-joining method^[31]^ .

### RNA isolation and quantitative real-time PCR analysis

Total RNA was extracted from young leaf, mature leaf, phloem, xylem, and root of the 'Danhong', 'Tongliao1', 84K poplar, and transgenic lines using an RNAprep Pure Plant Kit (TIANGEN, Beijing) according to the manufacturer's protocol. Total RNA was reverse-transcribed into cDNA using a TIANScript Ⅱ RT Kit (TIANGEN, Beijing). 'Danhong' and 'Tongliao1' lines were used to analyze the relative expression of poplar *PRE1* in young leaf, mature leaf, phloem, xylem, and root. The 84K poplar lines were used to analyze the expression pattern of *PRE1* in response to gibberellin (GA, 50 mg·L, Coolaber, Beijing) and salicylic acid (SA, 0.1 mM, Coolaber, Beijing) treatments.

qRT‒PCR was performed using a LightCycler480 real-time PCR system (Roche, Switzerland). Each reaction contained 10 μL of TB Green® Fast qPCR Mix Green Master (Takara, Beijing, China), 2 μL of primers (1 μL each of forward and reverse primers at a final concentration of 10 nM), 1 μL of diluted cDNA template and 7 μL of ddH_2_O. The *actin* gene (Potri.001G309500) was used as the reference gene, and the 2^−ΔΔCᴛ^ method was used to analyze the relative expression of poplar *PRE1* and other differentially expressed genes (DEGs) from the RNA-seq results. All experiments were performed in-triplicate (Supplemental Table S1).

### Gene cloning, vector construction, and plant transformation

The CDS of the *PsPRE1* gene was cloned from 'Tongliao1' cDNA *via* specific primers. The sequence was first cloned into the pDONR222 vector using BP Clonase (Gateway™ BP Clonase™ II Enzyme Mix, Invitrogen, Thermo Fisher Scientific, Shanghai, China) followed by subcloning into the binary expression vector pMDC32 using LR Clonase (Gateway™ BP Clonase™ II Enzyme Mix, Invitrogen, Thermo Fisher Scientific, Shanghai, China). Each vector was sequenced to ensure the *PsPRE1* gene did not contain any mutations (Sangon, Shanghai). The recombinant *pMDC32*-*PsPRE1* vector was transformed into *Agrobacterium tumefaciens* strain GV3101 using electroporation and strains containing the vector were verified using colony PCR. The Agrobacterium-mediated leaf disc method was used to transform 84K poplar. Transformed plants were selected on 1/2 MS (Phytotech, USA) medium containing 3 mg·L^−1^ hygromycin and 200 mg·L^−1^ Timentin. Successful transformants were verified by performing PCR on genomic DNA and qRT‒PCR on cDNA from transgenic plants^[32]^ (Supplemental Table S1).

### Salt stress and hormone treatments

Transgenic tissue culture seedlings (OE#14 and OE#15) were used for salt stress treatment. Comparable seedlings were selected and treated with 100 mM NaCl in 1/2 MS medium with at least three replicates per treatment. Plants were kept at 25 °C with a 16 h photoperiod and light intensity of 2,500 lux. After one month of treatment, morphological indicators were measured, and whole plant samples were collected. The samples were frozen in liquid nitrogen and stored at −80 °C.

84K poplar tissue culture seedlings of similar size were transplanted on 1/2 MS containing 50 mg·L^−1^ GA_3_ (Coolaber, Beijing), 50 mg·L^−1^ GA_4+7_ (Coolaber, Beijing), or 0.1 mM SA (Coolaber, Beijing) with three replicates per treatment. Plants were kept at 25 °C with a 16 h photoperiod and light intensity of 2,500 lux. After one month of treatment, three tissue samples were collected to analyze the expression pattern of poplar *PRE1* in response to gibberellin and salicylic acid.

### RNA-seq analysis of *PsPRE1*-overexpression lines and hybrid offspring

RNA-seq was performed on the roots of the *PsPRE1*-overexpression line OE#14. The roots of 30-day-old tissue culture seedlings were collected, frozen in liquid nitrogen, placed in a sterile 15 mL centrifuge tube, and sent to GENEDENOVO Biotechnology Co., Ltd. (Guangzhou, China) for RNA-seq analysis. Three biological replicates per line were analyzed with each replicate consisting of tissue from three seedlings pooled together.

'Danhong', 'Tongliao1', and offspring seedlings of similar size were selected for the RNA-seq analysis of drought and salt stress responses. The water content in the drought treatment group was adjusted to 55%−60% of the maximum water holding capacity of the perlite. The salt stress was applied by watering plants with 150 mM NaCl and maintaining a perlite water content of 100%. The control treatment was applied by watering plants with regular tap water and maintaining a perlite water content of 100%. After one month of growth, root tips were collected, frozen in liquid nitrogen, and sent to Biomarker Technologies Co., Ltd. (Beijing, China) for RNA-seq analysis. Three biological replicates per line were analyzed.

### Phenotype determination

Plant height, fresh weight above ground, maximum root length, total number of roots, and root fresh weight were quantified in the presence and absence of 100 mM NaCl treatment. At least three plants for each transgenic line were used to determine an average for each metric.

### Antioxidant enzyme activity analysis

After applying the control or 100 mM NaCl treatments, wild-type and transgenic lines were collected, and the enzymatic activity of superoxide dismutase (SOD), peroxidase (POD), catalase (CAT), and malondialdehyde (MDA) was quantified (Solarbio, Beijing) according to the instruction manual for experimental methods.

## Results

### Screening and expression pattern of *PRE1*

Previous QTL mapping by our group identified the poplar bHLH gene *PRE1* as being strongly linked with root dry weight trait (RDW) with a LOD value of 4.35. *PRE1* is 107.916 cM away from the marker QTL qRDW-LG18-21 in a hybrid offspring of 'Danhong' × 'Tongliao1'^[30]^ (Table 1). The root phenotypes of the two parents differed significantly. 'Tongliao1' exhibited significantly higher root dry weight and total root length compared to 'Danhong'^[30]^.

**Table 1:** QTL annotation of *PRE1*.

Trait	QTL code	LOD	Genetic distance	Candidate gene	Homologous gene	Chr	Phytozome annotation
Root Dry Weight	qRDW-LG18-21	4.35	107.916	Potri.004G128400	AT5G39860	Chr04	Similar to expressed protein in *Arabidopsis thaliana*; similar to supported by full length cDNA gi:26453215 from (*Arabidopsis thaliana*); [co-ortholog (2 of 2) of At1g26945]

**Figure 1:**
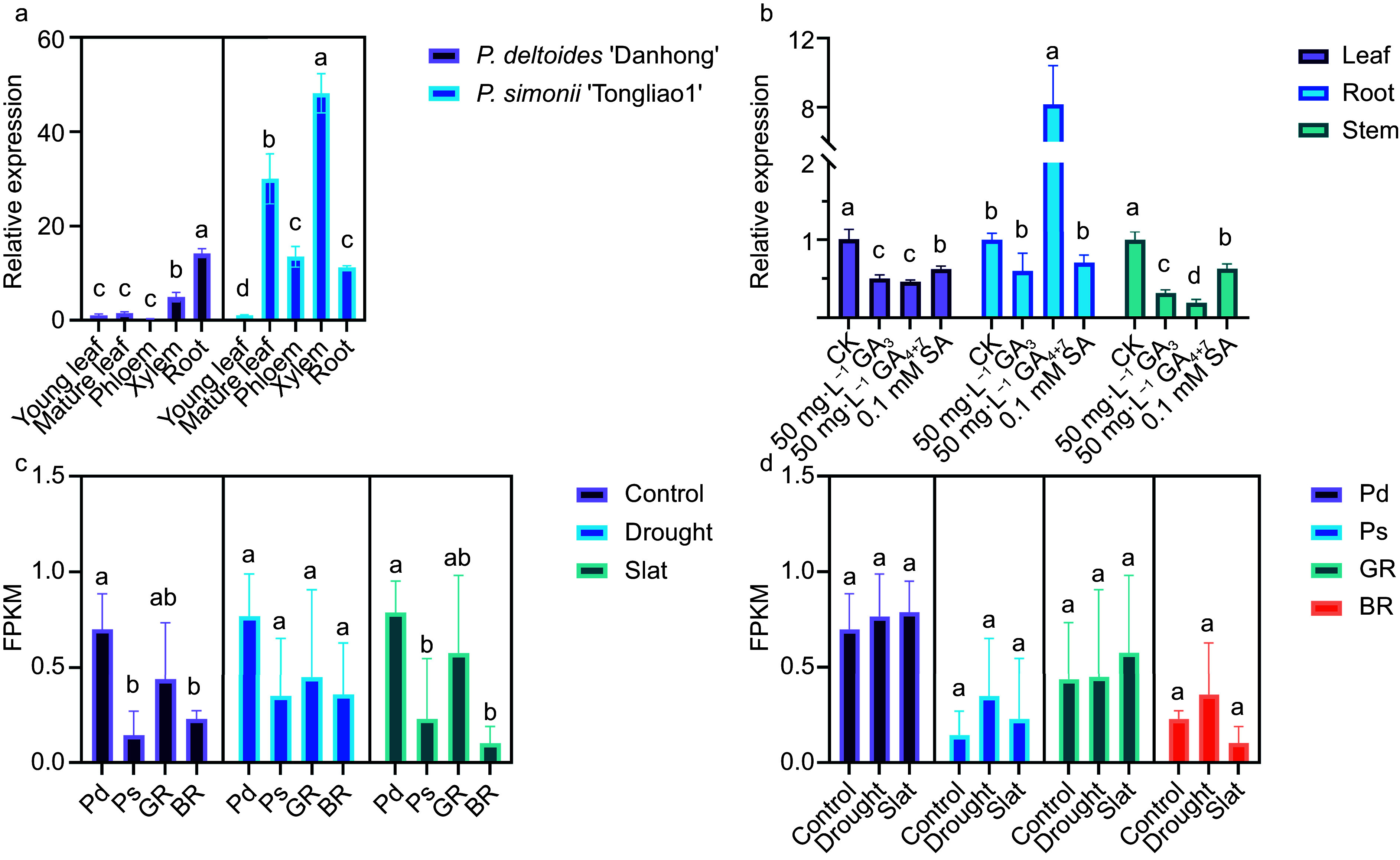
Tissue-specific expression patterns of poplar *PRE1* and hormone-induced expression changes. (a) Relative expression of poplar *PRE1* in various tissues. (b) Expression of poplar *PRE1* in response to salicylic acid and gibberellin. *actin* was used as a reference. (c) Poplar *PRE1* expression between parents and offspring. (d) Poplar *PRE1* expression in response to drought and salt stress. Pd: *Populus deltoides* 'Danhong', Ps: *Populus simonii* 'Tongliao1', GR: good rooting offspring, BR: bad rooting offspring. An ANOVA was used to determine statistical significance between sample means. Different letters above the bars indicate significantly different values (*p* < 0.05).

We further investigated the role of *PRE1* in poplar root development by determining its expression pattern in multiple tissues. The relative expression of *PRE1* in the xylem and root of 'Danhong' was significantly higher than its expression in other tissues. The relative expression of *PRE1* in the xylem of 'Tongliao 1' was also significantly higher than in most other tissues (Fig. 1a). However, in contrast to 'Danhong', the expression of *PRE1* in mature leaves of 'Tongliao 1' was much higher than its expression in other tissues except for the xylem (Fig. 1a). *PRE1* expression was relatively high in the root and xylem of both cultivars. This expression pattern is possibly related to the role of *PRE1* in poplar root and wood development.

Previous studies have shown that *PRE1* responds to gibberellin^[26]^. An analysis of *PRE1* gene expression in 84K poplar in response to hormone treatment revealed that *PRE1* was generally downregulated in leaf and stem tissue in response to gibberellin (Fig. 1b). In root tissue, *PRE1* expression was unaffected by GA_3_ treatments but increased approximately 8-fold in response to GA_4+7_ (Fig. 1b).

Previous studies have shown that *PRE1* promotes cell elongation^[27]^, which could lead to increased plant tolerance to stress. An RNA-seq analysis was performed on the parent lines ('Danhong' and 'Tongliao 1') and the offspring (F64 – good rooting (GR) and F75 – bad rooting (BR)) under drought and salt stress. *PRE1* expression was largely unaffected by drought or salt stress in all four of the lines. However, *PRE1* expression was generally higher in 'Danhong' and GR in control and treated plants, which suggests a possible positive role for *PRE1* in root development (Fig. 1c, Supplemental Table S2). *PRE1* expression was higher under drought and salt stress in both parent and offspring lines, revealing that poplar *PRE1* expression responds to these stresses (Fig. 1d). We ultimately chose to clone and further characterize *PsPRE1* from 'Tongliao1' because the rooting ability of this line was significantly better than that of 'Danhong'.

### *PsPRE1* is a bHLH transcription factor

**Figure 2:**
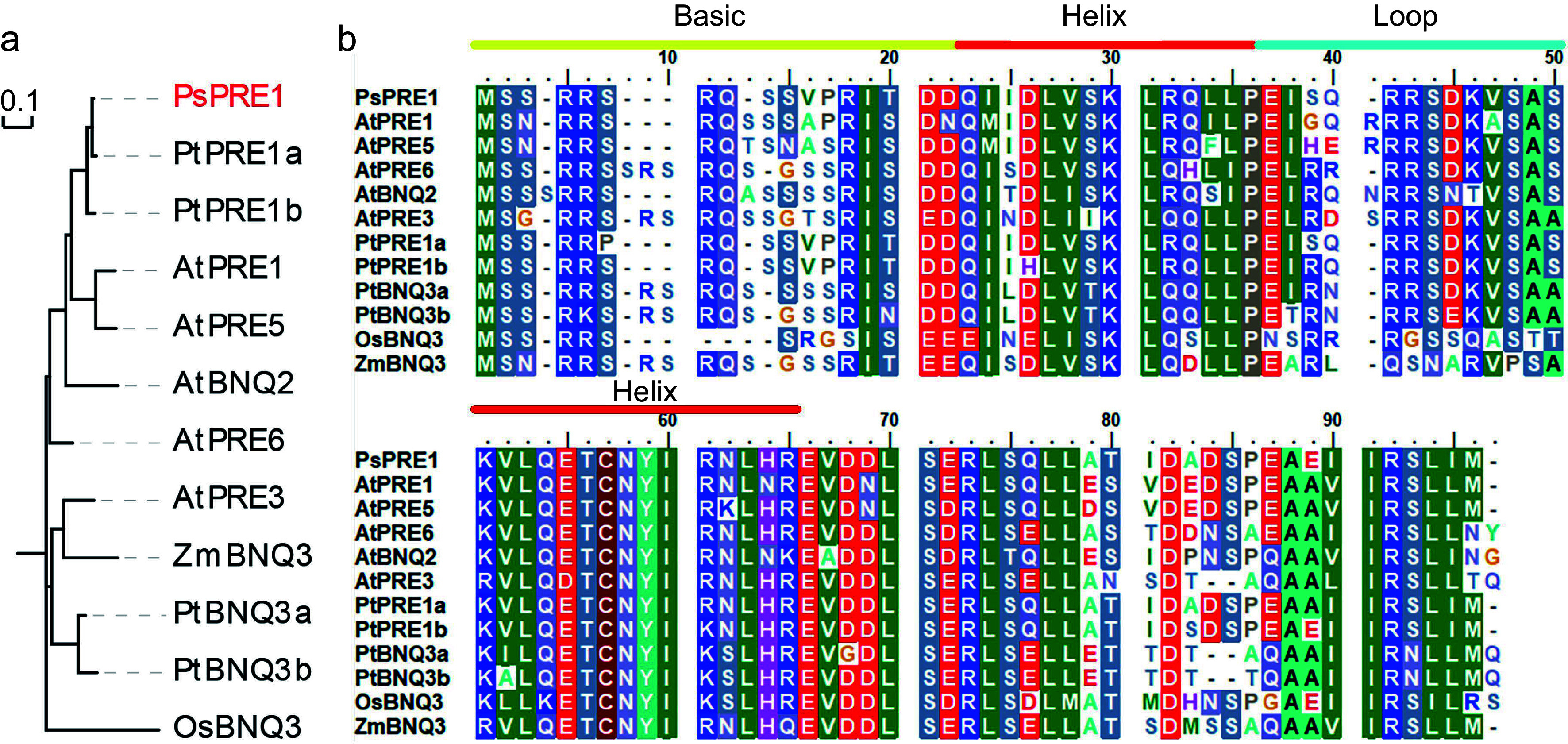
Phylogenetic comparison and protein sequence alignments of PRE1 homologs. (a) A phylogenetic tree containing PRE homologs from *Arabidopsis thaliana* (At), *Populus trichocarpa* (Pt), *Zea mays* (Zm), and *Oryza sativa* (Os) was constructed with PsPRE1 marked in red. (b) Protein sequences of the PRE homologs were aligned and conserved motifs were highlighted above the sequences.

A phylogenetic tree was constructed from the protein sequence of PsPRE1 and homologs in *Arabidopsis thaliana*, *Populus trichocarpa*, *Zea mays*, and *Oryza sativa*. The phylogenetic tree showed PsPRE1 is homologous to two PRE1 proteins from *P. trichocarpa* and one PRE1 protein from *A. thaliana* (Fig. 2a). PsPRE1 and its homologs belong to the bHLH family of proteins due to the presence of a conserved bHLH domain. An alignment of the PsPRE1 protein sequence with the PRE1 homologs revealed that the two helix regions within the bHLH domain were conserved, whereas the basic and loop regions were more variable (Fig. 2). bHLHs are often classified into typical and atypical bHLHs according to the number and type of amino acid residues in the basic region. Previous studies have demonstrated that Glu-13 and Arg/Lys-16 are required for bHLH transcription factors to bind to E-box elements^[19, 23]^. Interestingly, Glu-13 was not conserved in the basic region of the PRE1 proteins we analyzed. Therefore, PsPRE1 and its homologs were classified as atypical bHLHs (Fig. 2b).

### *PsPRE1* regulates the growth of poplar roots

Four *PsPRE1*-overexpression lines were obtained using Agrobacterium-mediated genetic transformation. The expression of *PsPRE1* in each line was confirmed to be higher than expression of *PsPRE1* in the wild-type background. Of the four overexpression lines, OE#14 had the highest expression of *PsPRE1* and OE#15 had an intermediate level of expression. These two lines were selected to further investigate the role of *PsPRE1* in root development (Supplemental Fig. S1).

**Figure 3:**
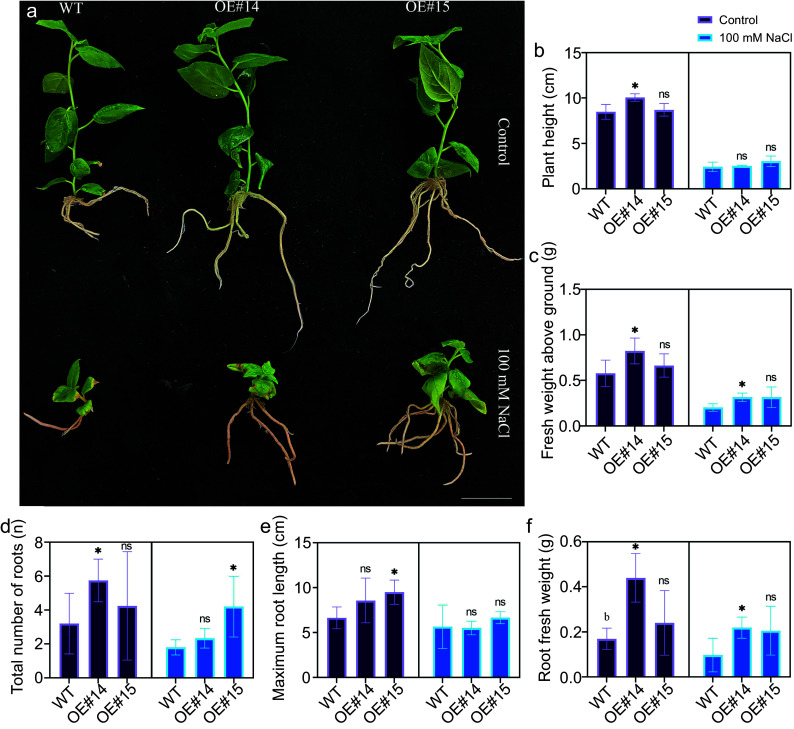
Overexpression of *PsPRE1* in poplar promoted root development and tolerance to salt stress. (a) Typical phenotypes of wild-type and the transgenic lines OE#14 and OE#15 overexpressing *PsPRE1* under control and salt stress conditions. Scale bar = 3 cm. (b) Plant height, (c) fresh weight above ground, (d) total number of roots, (e) maximum root length, and (f) root fresh weight of wild-type and transgenic lines under normal and salt stress conditions. A Student's *t*-test was used to determine statistical significance between sample means. *, *p* < 0.05; ns, nonsignificant.

In normal growth medium, the roots of the *PsPRE1*-overexpression lines grew longer and were more abundant than the roots of wild-type plants (Fig. 3a, d & e). The plant height, fresh weight aboveground, and root fresh weight of OE#14 were significantly higher than wild-type plants (Fig. 3b, c & f). Only the maximum root length of OE#15 was significantly higher than wild-type plants (Fig. 3e). Overall, our data demonstrated that overexpression of *PsPRE1* promotes root development in non-stress conditions.

### Overexpression of *PsPRE1* increases tolerance to salt stress

The OE#14 and OE#15 transgenic lines grew better than wild-type plants under salt stress treatments according to multiple physiological metrics (Fig. 3). The fresh weight aboveground and root fresh weight traits of OE#14 were significantly higher than wild-type plants upon treatment with 100 mM NaCl, whereas the total root number of OE#15 was significantly higher than wild-type plants (Fig. 3). These data suggest that *PsPRE1* is a positive regulator of salt stress tolerance in poplar.

To better understand the mechanism behind increased salt stress tolerance in the *PsPRE1-*overexpression lines, the expression and enzymatic activity of superoxide dismutase (SOD), peroxidase (POD), catalase (CAT), and malondialdehyde (MDA) were assessed. These enzymes are involved in the detoxification of reactive oxygen species (ROS), which accumulate in plants subjected to salt stress^[33]^. The expression of these changes was unaffected by salt treatment in both the wild-type and *PsPRE1*-overexpression lines (Supplemental Fig. S2). However, we did observe elevated CAT enzymatic activity in the *PsPRE1*-overexpression lines compared to the wild-type lines under both normal and salt stress conditions (Supplemental Fig. S2).

### RNA-seq of the *PsPRE1*-overexpression line

**Figure 4:**
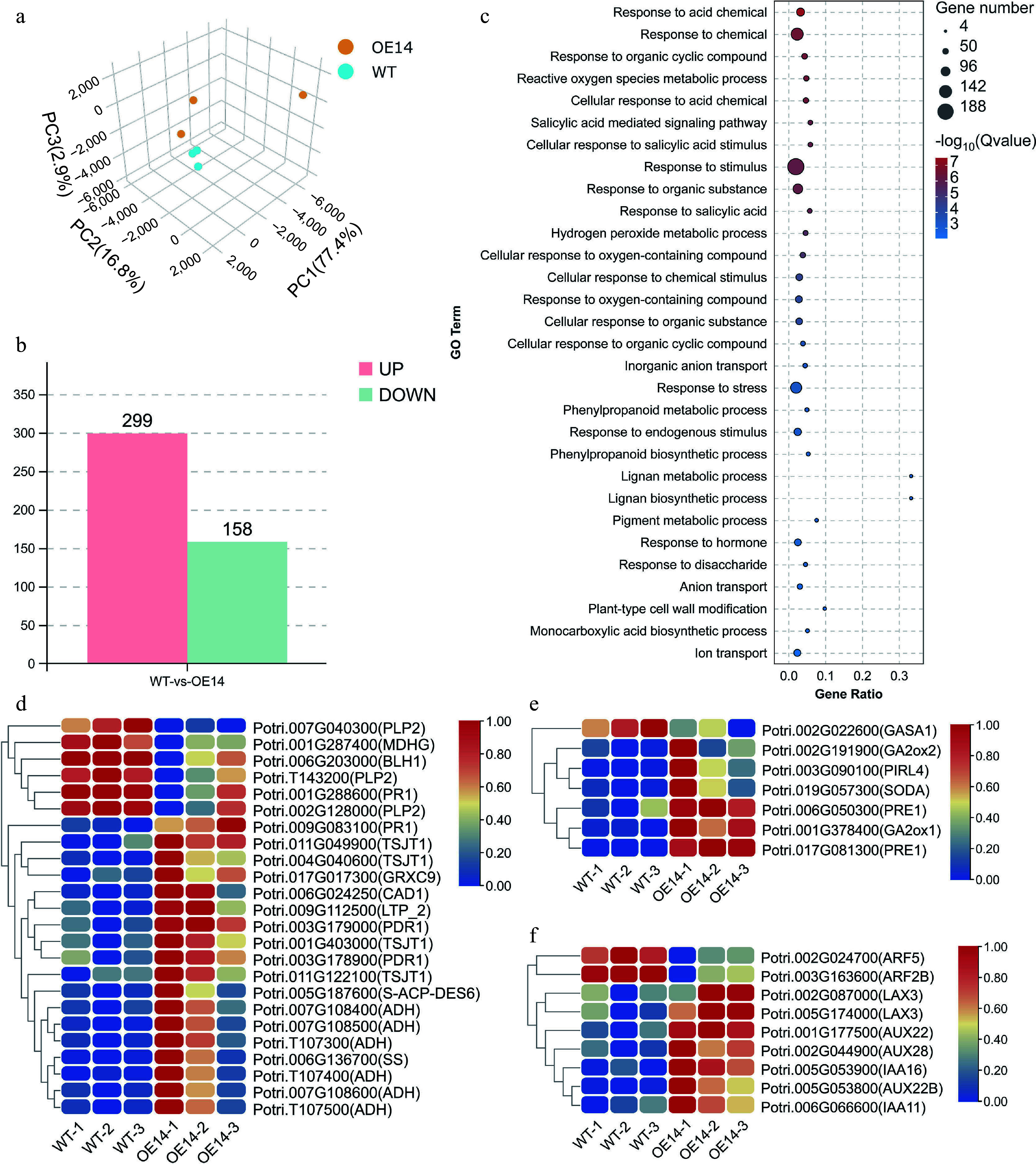
Identifying differentially expressed genes (DEGs) in transgenic poplar line OE#14 overexpressing *PsPRE1*. (a) A principal component analysis (PCA) of the expressed genes revealed a clear separation between samples with principal component 1 (PC1), PC2, and PC3 explaining 77.4%, 16.8%, and 2.9% of the total variance, respectively. (b) The number of DEGs in wild-type (WT) versus the OE#14 line. Up-regulated DEGs are in pink and down-regulated DEGs are in light green. (c) Gene Ontology (GO) enrichment analysis of DEGs. The node color represents the -log10 transformed false discovery rate (FDR) corrected p-value. The node size represents the number of DEGs within each GO term. Heatmap summarizing the expression of DEGs related to (d) salicylic acid, (e) gibberellin, and (f) auxin from the GO enrichment results.

Based on the above experiments, we demonstrated that *PsPRE1* promotes root development. To better understand the mechanism of *PsPRE1*-promoted root development, we performed an RNA-seq analysis on the roots of the OE#14 *PsPRE1*-overexpression line. A total of 457 differentially expressed genes (DEGs) between the OE#14 and wild-type lines were identified using RNA-seq. Of the DEGs identified, 299 had higher expression in OE#14 and 158 had lower expression in OE#14 (DESeq2, FDR < 0.05, fold change ≥ 2) (Fig. 4b). A Gene Ontology (GO) enrichment analysis revealed that three GO terms (response to chemical, response to stimulus, and response to stress) contained the largest number of DEGs. These results support the results from our salt stress analysis of the *PsPRE1*-overexpression lines and further suggest that *PsPRE1* might be involved in poplar responses to stress (Fig. 4c).

**Figure 5:**
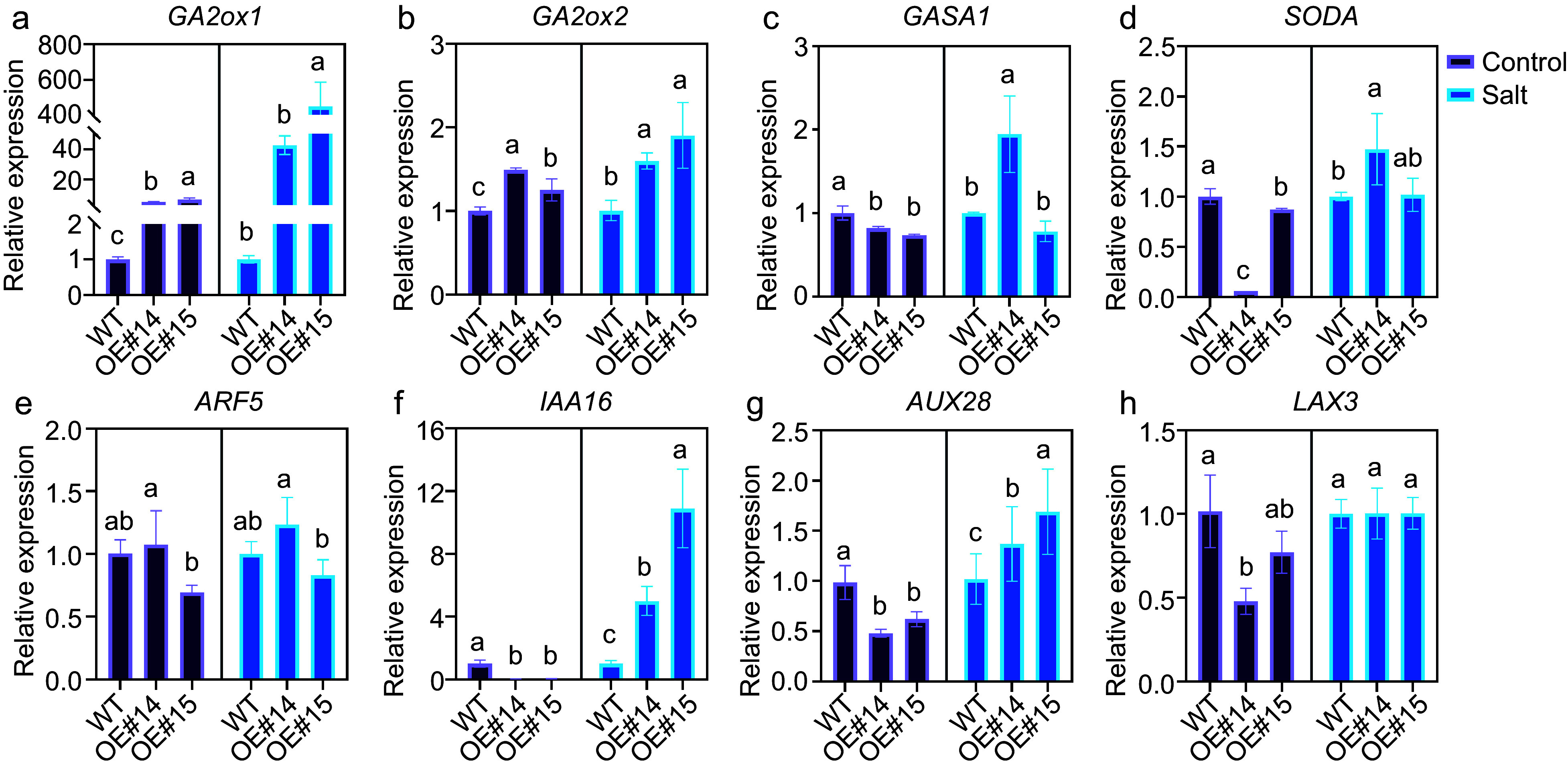
Auxin- and gibberellin-related genes expression in the *PsPRE1*-overexpression lines. *actin* was used as a reference. Statistical analysis based on ANOVA, different letters above the bars indicate significantly different values (*p* < 0.05).

GO terms related to plant hormones were also present among the DEGs. Three enriched GO terms were related to salicylic acid, and seven and nine DEGs were related to gibberellin and auxin, respectively. These results support our hypothesis that *PsPRE1* may be involved in the regulation of these hormones (Fig. 4c). We performed a more comprehensive analysis of the expression of genes involved in the auxin-, gibberellin-, and salicylic acid-related pathways. Clear differences in gene expression were observed between wild-type and OE#14 plants in these hormone pathways (Fig. 4d−f). We demonstrated that poplar *PRE1* was downregulated in leaf, root, and stem tissue in response to salicylic acid (Fig. 1b). The expression of auxin- and gibberellin-related genes after 100 mM NaCl treatment was verified using qRT‒PCR. Increased expression of *GA2ox1* and *GA2ox2* after salt treatment may be a means of reducing gibberellin levels in plants and eliminating the inhibitory effect of gibberellin on root development^[14]^. In addition, the expression of some auxin-related genes was also increased to varying degrees (Fig. 5) . The results were consistent with the overexpression lines phenotype and RNA-seq results. The results also suggested that *PsPRE1* might regulate root development by regulating the expression of auxin-, gibberellin-, and salicylic acid-related genes.

## Discussion

Previous studies have demonstrated that some bHLH transcription factors regulate plant growth in response to environmental stresses such as drought and salt^[34−37]^. Salt stress is one of the most prevalent environmental stresses worldwide^[38]^. Identifying and characterizing conserved regulators of plant stress responses could lead to improved crop varieties with better tolerance of saline soils and enhanced crop yields in these challenging environments. In this study, we identified *PsPRE1* as a positive regulator of poplar drought and salt stress tolerance. *PsPRE1* is a member of the bHLH family with an atypical basic region and lacks critical residues required for binding to E-box cis-elements (Fig. 2b)^[23]^. Previous studies have shown that HLH4 is able to bind to CIB5 and prevent CIB5 from promoting the expression of *EXPs*. PRE1 suppresses HLH4, which leads to the activation of CIB5 and promotes cell elongation^[28, 39]^. We identified seven *EXPs* that were differentially expressed in the *PsPRE1*-overexpression line, suggesting that *PsPRE1* may regulate root development by regulating the expression of *EXPs* (Supplemental Table S3). Subsequent experiments will focus on further characterizing the regulatory role of PsPRE1 on *EXP* genes expression.

Gibberellins are plant hormones that play essential roles in seed germination, organ elongation and expansion, trichome development, the transition from vegetative to reproductive growth, and numerous other physiological functions^[40−42]^. While many gibberellins have been identified, most of them are precursors of bioactive gibberellins or deactivated metabolites. It is generally accepted that only GA_1_, GA_3_, GA_4_, and GA_7_ are active hormones^[43]^. In plants, *PRE* genes exhibit differential responses to gibberellins. For example, Arabidopsis *PRE1* regulates hypocotyl development in response to gibberellin^[26, 39, 44]^. Our results demonstrated that *PsPRE1* expression in poplar roots is induced by GA_4+7_. In *A. thaliana*, GIBBERELLIN INSENSITIVE1 (GAI1) is a DELLA protein that functions as a negative regulator of upstream gibberellin signaling. Gibberellin-dependent induction of *PRE1* is decreased in the *gai*-*1* mutant^[26]^, which suggests that *PsPRE1* may be involved in the regulation of the gibberellin regulatory pathway. Gibberellin is also involved in the regulation of salt stress in plants. Two GA metabolism-related genes, *AtGA2ox7* and *OsGA2ox5*, support plant survival by inhibiting plant growth^[38, 45, 46]^ during salt stress.

Salicylic acid is an important regulator of plant root development that can promote the growth of adventitious roots^[47]^. We found that the *PsPRE1*-overexpression line had multiple DEGs involved in the salicylic acid pathway. There is also a known regulatory relationship between salicylic acid and auxin. Salicylic acid inhibits the expression of *PIN4* and *PIN7* but induces the expression of *TAA1*. The concentration of salicylic acid also affects the expression pattern of *PIN1*^[48]^. The auxin-related genes *LAX3*, *Auxin Response Factor2* (*ARF2*), and *IAA16*^[49−51]^ were up-regulated in the *PsPRE1*-overexpression line, suggesting that adventitious root development may be promoted by *PsPRE1* through increased plant response to and translocation of auxin. In summary, *PsPRE1* may regulate root development in poplar through multiple hormone pathways.

Sax first described the concept of a QTL when he observed the segregation of seed weight associated with the segregation of a seed coat color marker in green bean (*Phaseolus vulgaris* L.)^[52]^. He discovered that one gene controlling seed color could be associated with one or more polygenes controlling seed size^[52]^. With the development of high throughput sequencing technologies, mapping gene linkage maps by sequencing rapidly speeds up the QTL mapping process^[53]^. Our research group previously used two poplar varieties ('Danhong' and 'Tongliao1') with multiple phenotypic differences to obtain multiple genes associated with agronomic traits^[30]^. Among the genes identified, this study demonstrates that the poplar *PRE1* gene is associated with root dry weight traits, suggesting *PRE1* may regulate poplar root development. We cloned *PsPRE1* from 'Tongliao1', overexpressed it in 84K poplar, and demonstrated that the overexpression of *PsPRE1* promotes root development and improves salt tolerance. These results are consistent with the QTL results and demonstrate that QTL mapping is a reliable molecular-assisted breeding method.

## Conclusion

Based on QTL results, poplar *PRE1* was selected as a candidate gene associated with the root dry weight trait. *PsPRE1* was cloned from 'Tongliao1' and overexpressed in 84K poplar. Overexpression of *PsPRE1* promoted adventitious root development and enhanced growth under salt stress. An RNA-seq analysis of root tissue in the transgenic line demonstrated that several differentially expressed genes were related to auxin-, gibberellin-, and salicylic acid-related pathways. These results suggest that the regulation of root development in poplar by *PsPRE1* may involve multiple hormones.

## SUPPLEMENTARY DATA

Supplementary data to this article can be found online.

## Data Availability

The raw sequence data reported in this paper have been deposited in the Genome Sequence Archive^[54]^ at the National Genomics Data Center^[55]^, China National Center for Bioinformation / Beijing Institute of Genomics, Chinese Academy of Sciences (GSA: CRA007914 and CRA008053) and are publicly accessible at https://ngdc.cncb.ac.cn/gsa.
