# Supplementary figures and images for: PsPRE1 is a basic helix-loop-helix transcription factor that confers enhanced root growth and tolerance to salt stress in poplar
Source: For Res (Fayettev). 2023 Jun 29;3:16. doi: 10.48130/FR-2023-0016 (PMC11524248; doi:10.48130/FR-2023-0016)

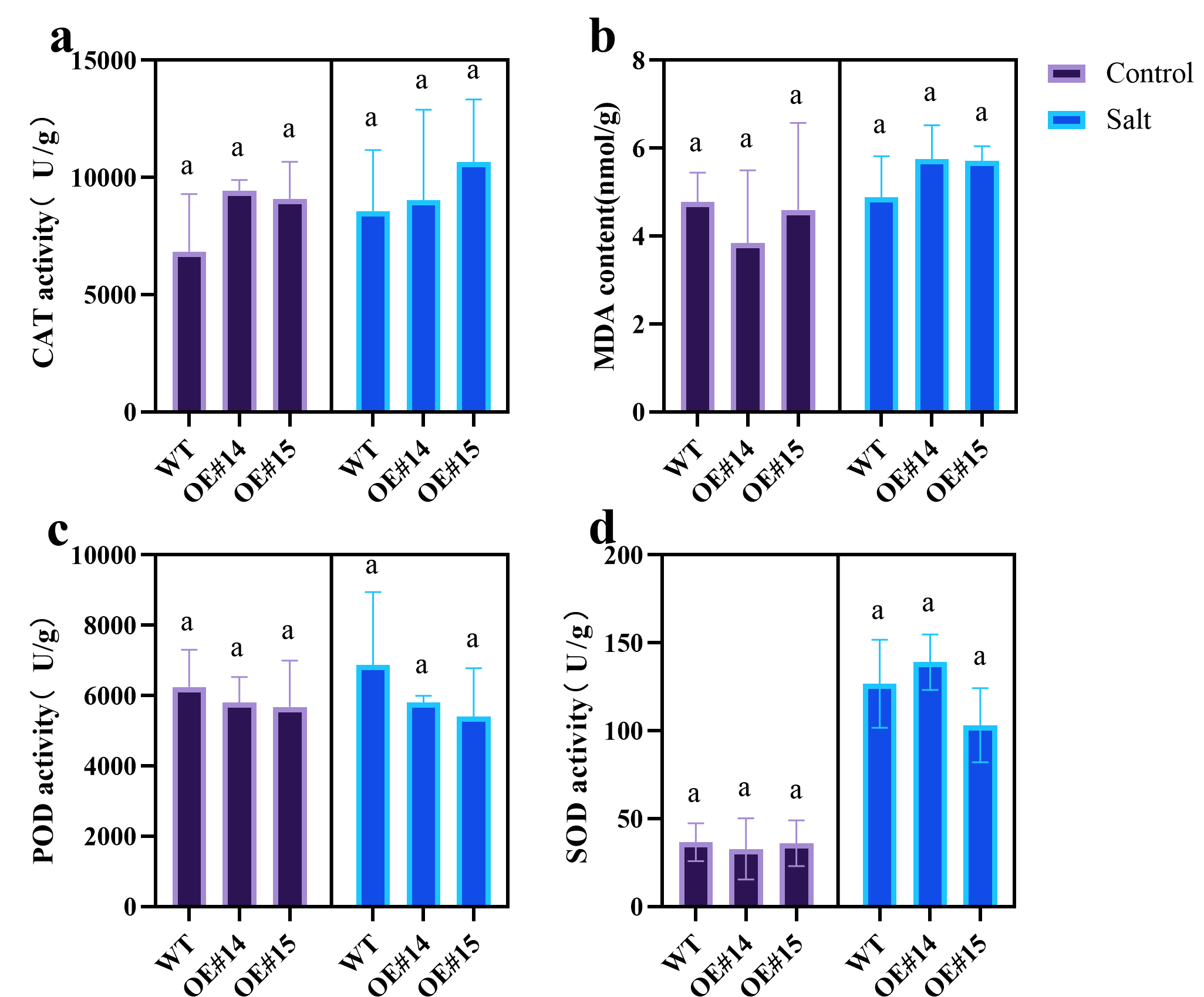

Supplement: Supplementary file 1 — Supplementary data to this article can be found online. [file FR-2023-0016-S1.zip › 10.48130_FR-2023-0016-Suppl-FigureS2.jpg]

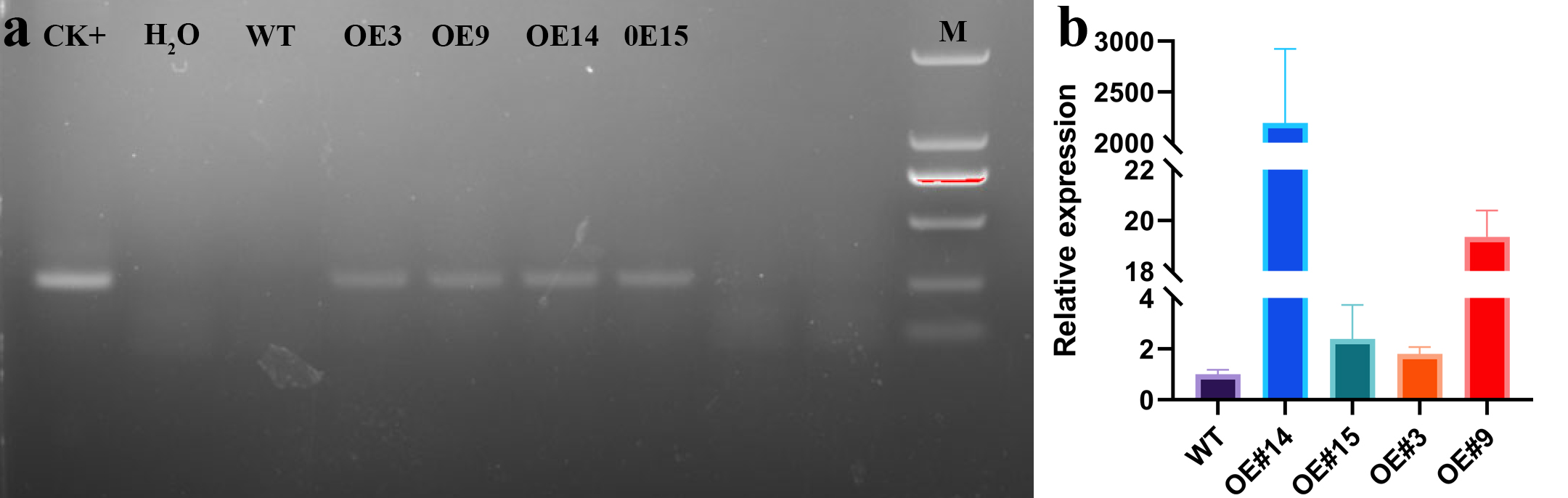

Supplement: Supplementary file 1 — Supplementary data to this article can be found online. [file FR-2023-0016-S1.zip › 10.48130_FR-2023-0016-Suppl-FigureS1.jpg]
